# Intravesical Platelet-Rich Plasma Injection for Refractory Interstitial Cystitis/Painful Bladder Syndrome: A Systematic Review and Meta-analysis

**DOI:** 10.1007/s00192-026-06515-9

**Published:** 2026-04-06

**Authors:** Gabriel Chahade Sibanto Simões, Caio de Oliveira, Lucas Mira Gon, Cássio Luís Zanettini Riccetto, Gabriel Chahade Sibanto Simões

**Affiliations:** State University of Campinas (UNICAMP), Campinas, SP Brazil

**Keywords:** Interstitial Cystitis, Platelet-Rich Plasma, Intravesical Administration

## Abstract

**Introduction and Hypothesis:**

Intravesical injection of platelet-rich plasma (PRP) has emerged as a novel therapeutic approach for patients with interstitial cystitis/ bladder pain syndrome (IC/BPS). This systematic review and meta-analysis aim to evaluate the efficacy of PRP in alleviating pain and other related symptoms and outcomes in women.

**Methods:**

This systematic review and meta-analysis followed PRISMA guidelines and the PICO framework. A comprehensive search was conducted in multiple databases. Risk of bias was assessed using the Cochrane Review Manager (RevMan), and quality of evidence was evaluated using the Modified Oxford Centre for Evidence-Based Medicine tool. All included studies were quasi-experimental, uncontrolled, and non-randomized, resulting in a low overall level of evidence**.** A pre–post meta-analysis was performed assessing outcomes such as Global Response Assessment (GRA), pain (VAS), symptom scores (ICSI, ICPI, OSS), urinary frequency, nocturia, and urodynamic parameters. Standardized mean differences were calculated using a random-effects model, and heterogeneity was assessed using Cochran’s Q test and the I^2^ statistic.

**Results:**

The meta-analysis revealed a potential treatment effect, with a mean success rate (GRA ≥ 2) of 48%. Given that all the results derive from uncontrolled and non-randomized studies, they should therefore be interpreted with caution. Statistically significant improvements were observed in pain scores, urinary frequency, nocturia, and bladder function, including increases in Qmax and voided volume.

**Conclusions:**

Intravesical PRP injection may represent a promising therapeutic option for refractory IC/BPS, showing trends toward improvement in pain, nocturia, frequency, and bladder capacity. However, as current evidence is derived exclusively from uncontrolled and non-randomized studies, its efficacy remains uncertain and the findings should be interpreted with caution. Nonetheless, higher-quality randomized controlled trials are required to confirm its efficacy and better define its role in clinical practice.

**Supplementary Information:**

The online version contains supplementary material available at 10.1007/s00192-026-06515-9.

## Introduction

Interstitial cystitis/bladder pain syndrome (IC/BPS) is a chronic and debilitating condition with a significant impact on quality of life. It is clinically diagnosed and characterized by pelvic pain lasting more than six weeks, often associated with bladder distension, urgency, and increased urinary frequency [1].

Although the pathophysiology of IC/BPS is not fully understood, urothelial dysfunction plays a central role. This involves disruption of the glycosaminoglycan layer, chronic inflammation, excessive urothelial apoptosis, mast cell activation, and upregulation of nociceptive receptors. Persistent inflammation and impaired urothelial regeneration lead to increased bladder permeability, pain, and irritative symptoms. Enhancing the regenerative capacity of urothelial progenitor cells is thought to restore a healthier epithelium and alleviate symptoms [2–4].

Platelets, beyond their role in hemostasis, contribute to tissue repair and regeneration through the release of growth factors and anti-inflammatory cytokines. Platelet-rich plasma (PRP), a concentrate rich in these components, promotes epithelial proliferation, differentiation, and wound healing, while modulating inflammatory responses and reducing neuropathic pain. PRP is already widely used in orthopedics, dermatology, and ophthalmology [5–7].

Intravesical PRP injections have recently been proposed as a novel approach for managing refractory IC/BPS cases, with preliminary studies showing promising results. Given the chronic nature of IC/BPS and the limitations, high cost, and variable efficacy of current therapies—including lifestyle changes, hydrodistension, anti-inflammatory and immunosuppressive agents, hyaluronic acid instillations, and botulinum toxin—PRP may represent a viable and innovative alternative [8, 9].

This study aimed to conduct a systematic review and meta-analysis to evaluate the efficacy and safety of intravesical PRP injections for treating women with refractory IC/BPS.

## Materials and Methods

**Fig. 1:**
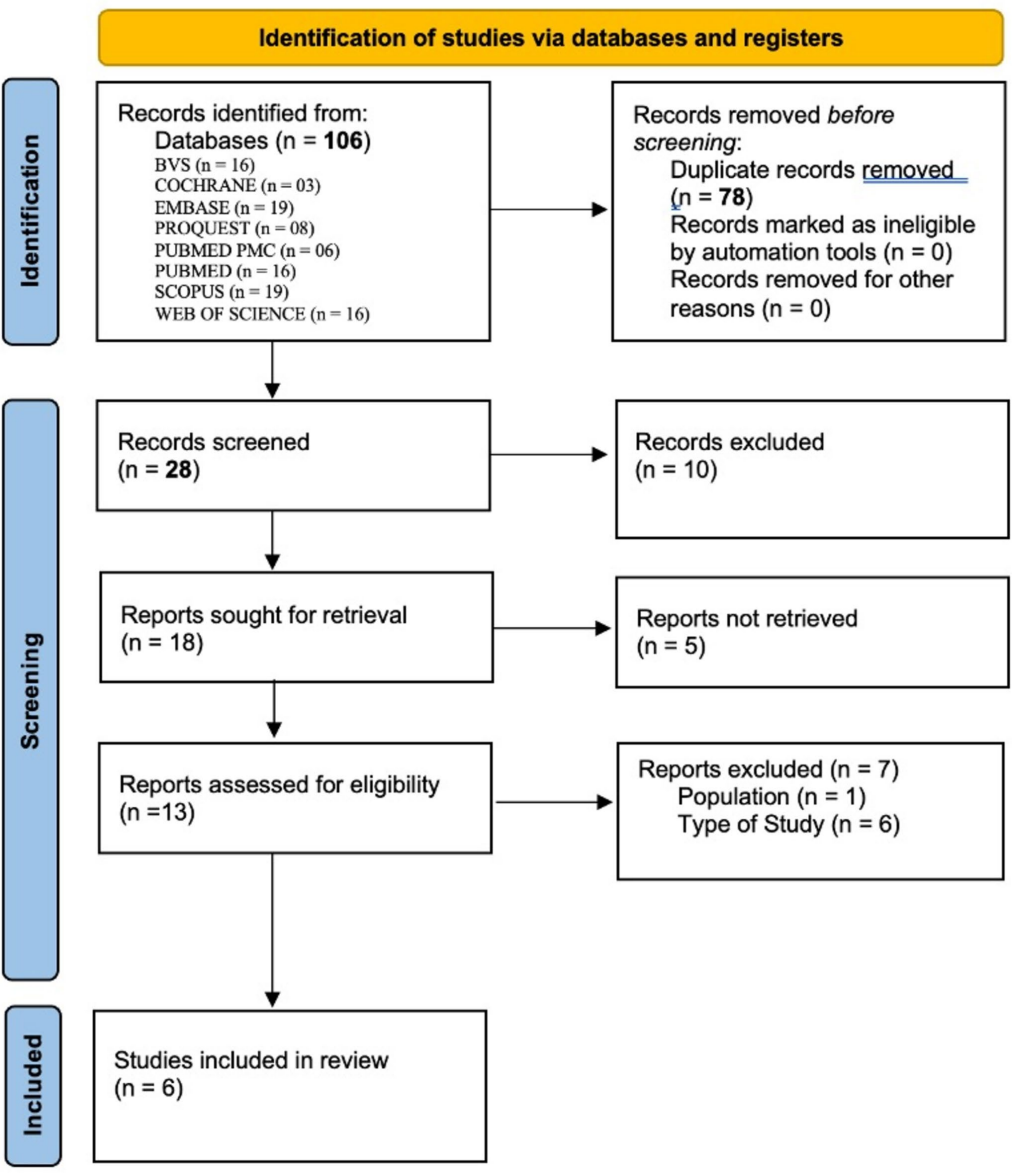
PRISMA 2020 flow diagram illustrating the literature search and selection process for studies included in the systematic review and meta-analysis

This systematic review followed the *Preferred Reporting Items for Systematic Reviews and Meta-Analyses (PRISMA)* guidelines, as updated by Page et al. in 2020 (Fig. 1) [10]. The research question was developed using the PICO framework: *Population* – women with IC/BPS refractory to conventional treatments; *Intervention* – intravesical injection of platelet-rich plasma (PRP); *Comparison* – pre- and post-treatment clinical parameters; and *Outcome* – treatment effectiveness based on subjective scores and objective measures.

A comprehensive electronic search was conducted in July 2023 across multiple databases, including MEDLINE/PubMed, PMC/PubMed, Web of Science, Embase, Scopus, LILACS/BVS, ProQuest, and the Cochrane Library. A Search strategy was developed, using the following terms: "Cystitis, Interstitial" AND "Platelet-Rich Plasma" AND "Administration, Intravesical" (detailed in Supplementary File 2). This review was registered with PROSPERO (ID: CRD42024519553). Ethical approval was waived (Research Ethics Committee statement: OF. CEP/PRP/Nº 064/2024).

Inclusion criteria encompassed studies published up to July 2023, in any language or country, evaluating the use of intravesical PRP for IC/BPS in women, comparing pre- and post-treatment outcomes. Exclusion criteria included: (1) animal studies; (2) case reports; (3) literature reviews, systematic reviews, meta-analyses, case series, book chapters, expert opinions, editorials, interviews, seminars, posters; and (4) unpublished or incomplete studies lacking extractable data.

Study selection and data extraction were performed independently by two reviewers (GCSS and CO). Discrepancies were resolved by consensus or a third senior reviewer (LMG). Extracted data included: authorship, publication year, study design, sample characteristics and size, intervention protocols, subjective and objective outcomes, treatment success criteria, and reported complications.

### Risk of Bias and Evidence Quality

Risk of bias was assessed using the Cochrane Review Manager (RevMan) by two independent reviewers. The quality of evidence was rated using the modified Oxford Centre for Evidence-Based Medicine Levels of Evidence (LoE).

### Meta-Analysis

A meta-analysis was performed to evaluate the effects of PRP in a single-group design (pre- and post-intervention). The standardized mean difference (SMD = post – pre) was calculated using a random-effects model. Proportions and mean differences with 95% confidence intervals (CI) were estimated using random-effects linear models. Heterogeneity was assessed using Cochran’s Q and Higgins & Thompson’s I^2^ statistics. Results are presented in forest plots. All available data from eligible studies were included in the quantitative synthesis. When specific outcome parameters were missing or incompletely reported, those variables were excluded from the corresponding comparison, while the remaining data from the same study were retained for analysis.

Evaluated outcomes included subjective scores (shown in the supplementary file): Global Response Assessment (GRA), Visual Analog Scale (VAS), O’Leary-Sant Symptom Score (OSS), Interstitial Cystitis Symptom Index (ICSI), and Interstitial Cystitis Problem Index (ICPI); as well as objective measures: urinary frequency, nocturia, and urodynamic parameters such as functional bladder capacity (FBC), maximum flow rate (Qmax), voided volume, post-void residual (PVR), and cystometric bladder capacity (CBC). Treatment success was defined as an improvement of ≥ 2 points in GRA between baseline and post-treatment assessments.

## Results

### Study Selection and Characteristics

A total of 106 records were initially identified, with 78 duplicates removed. After title and abstract screening, 18 full texts were assessed. Of these, 12 were excluded (7 did not meet eligibility criteria, 5 were unavailable in full), resulting in 6 studies included in the systematic review and meta-analysis (Fig. 1).

All included studies were non-randomized, uncontrolled prospective trials involving women with refractory IC/BPS. The total sample comprised 182 patients, with individual studies ranging from 15 to 52 participants. PRP was prepared using a two-step centrifugation method from 50 mL of whole blood and injected suburothelially under general anesthesia in four sessions spaced one month apart. The GRA scale was used as the primary outcome in five studies, with treatment success defined as a GRA score ≥ 2 at 1–3 months post-treatment. The GRA is a widely used patient-reported measure in IC/BPS studies, though formal validation has not been reported in all populations. Study characteristics and levels of evidence (all classified as 2 C) are summarized in Table 1.

**Table 1:** Levels of evidence

Study	Level of Evidence (OCEBM)
Jhang et al. 2017 [11]	2C
Jhang et al. 2019 [8]	2C
Jiang et al. 2022 [12]	2C
Lee et al. 2022. [13]	2C
Jhang et al. 2022. [14]	2C
Jhang et al. 2023 [15]	2C

### Risk of Bias and Quality Assessment

According to the Oxford Centre for Evidence-Based Medicine, all studies were rated as level 2 C evidence. Due to their quasi-experimental design, risk of detection bias was high, with no randomization or blinding. However, attrition and reporting biases were considered low. Full risk assessments are shown in figures of the supplementary file.

### Primary Outcomes: Global Response Assessment

Treatment success differed between studies, ranging from 26.3% (Jhang et al. 2022) to 67.5% (Jhang et al. 2019). Results are described in Table 2.

**Table 2:** Percentage of patients with therapeutic success at the end of treatment

Study	GRA ≥ 2 at the end of treatment	Mean GRA + standard deviation at the end of treatment
Jhang et al. 2017	54%	1.6 ± 0.9
Jhang et al. 2019	67,5%	1.94 ± 0,93
Lee et al. 2022	42,3%	1,35 ± 1,06
Jhang et al. 2022	26,3%	1,11 ± 0,66
Jhang et al. 2023	46,7%	1,53 ± 1,20

**Fig. 2:**
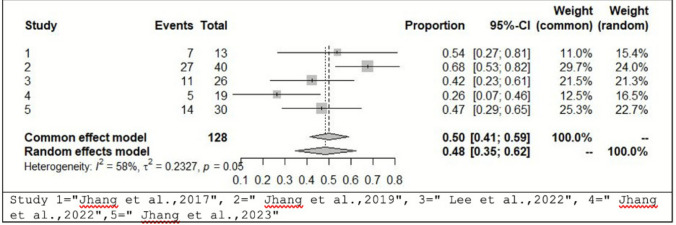
Forest plot representing the proportion of patients with treatment success (Global Response Assessment [GRA] ≥ 2) after intravesical PRP injections. Pooled analysis was performed using a random-effects model with 95% confidence intervals

**Fig. 3:**
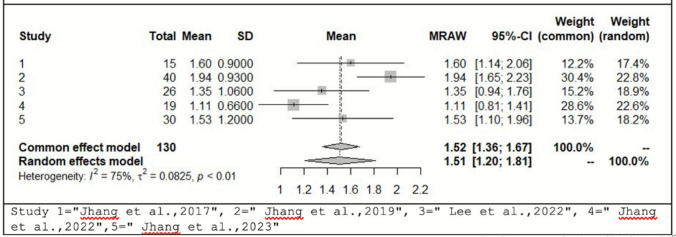
Forest plot showing the pooled mean GRA score at the end of treatment across five studies. The analysis used a random-effects model. Higher GRA scores reflect better clinical response

The meta-analysis of five studies (n = 128) showed a treatment success rate of 48.3% (95% CI: 34.7–62.2%; I^2^ = 58%) (Fig. 2). The pooled mean GRA score after treatment was 1.51 (95% CI: 1.20–1.81; I^2^ = 75%) (Fig. 3).

### Symptom Scores

The pre- and post-treatment results of the scores and clinical parameters evaluated are described in Table 3. Regarding the symptom scores, the meta-analysis showed improvement in all of them, as shown in figures in the supplementary file and described below:

**•ICSI**: Mean reduction = –0.93 (95% CI: –1.12 to –0.74; I^2^ = 12%; p < 0.0001)
**•ICPI**: Mean reduction = –1.00 (95% CI: –1.35 to –0.64; I^2^ = 72.5%; p < 0.0001)
**•OSS**: Mean reduction = –1.01 (95% CI: –1.33 to –0.70; I^2^ = 58%; p < 0.0001)
**•VAS (pain)**: Mean reduction = –0.63 (95% CI: –0.92 to –0.34; I^2^=66.5%; p < 0.001)

**Table 3:** Pre- and post-treatment results of the scores and clinical parameters evaluated

Study	Jhang et al. 2017		Jhang et al. 2019		Jiang et al. 2022		Lee et al. 2022		Jhang et al. 2022		Jhang et al. 2023	
Parameter	Pre	Post	Pre	Post	Pre	Post	Pre	Post	Pre	Post	Pre	Post
VAS	3.7 ± 1.8	2.6 ± 2.9	3.38 ± 2.89	1.10 ± 1.85	4.1 ± 3.1	1.4 ± 2	4.64 ± 3.59	2.86 ± 2.54	4.05 ± 3.27	1.95 ± 2.34	3.57 ± 3.14	3.17 ± 3.18
ICPI	9.7 ± 3.6	6.5 ± 4.6	9.80 ± 3.6	4.23 ± 3.46	10.6 ± 4.0	4.8 ± 3.9	10.7 ± 3.37	8.7 ± 3.77	9.47 ± 4.17	6.58 ± 3.5	11.63 ± 3.21	8.6 ± 4.06
ICSI	9.7 ± 4.2	8.0 ± 4.1	9.04 ± 3.72	10.2 ± 4.9	5.5 ± 3.6	11.60 ± 4.22	8.20 ± 3.16	8.20 ± 3.16	11.5 ± 4.86	7.95 ± 3.36	12.57 ± 4.43	8.33 ± 4.4
OSS	19.3 ± 7.2	14.7 ± 8.6	19.1 ± 6.95	9.26 ± 6.33	20.4 ± 8.8	17.3 ± 5.64	22.30 ± 6.72	17.30 ± 5.64	21 ± 8.37	14.7 ± 6.37	-	-
Frequency	14.2 ± 9.2	9.2 ± 3.5	14.6 ± 10.3	9.97 ± 3.42	13.1 ± 7.3	9.9 ± 3.4	21.4 ± 13.35	14.47 ± 9.99	-	-	11.7 ± 6.39	10.95 ± 4.08
Nocturia	-	-	2.6 ± 1.32	1.97 ± 1.33	2.5 ± 1.3	2.0 ± 1.4	4.17 ± 3.03	2.9 ± 1.91	-	-	3.28 ± 1.59	2.35 ± 1.8
FBC (ml)	199 ± 126	325 ± 175	291 ± 134	335 ± 98	298 ± 131	336 ± 103	202 ± 129	261 ± 156	217 ± 116	290 ± 124	260 ± 127	305 ± 136

### Functional Bladder Parameters

The meta-analysis demonstrated significant improvement in bladder functional parameters, as shown in figures in the supplementary file and described below:

**•Urinary frequency**: Mean reduction = –0.45 (95% CI: –0.61 to –0.29; I^2^ = 0%; p < 0.0001)

**•Nocturia**: Mean reduction = –0.45 (95% CI: –0.62 to –0.28; I^2^ = 0%; p < 0.0001)

**•Functional bladder capacity (FBC)**: Mean increase = 0.41 (95% CI: 0.26 to 0.56; I^2^ = 0%; p < 0.0001)

### Urodynamic Parameters

The results of the urodynamic parameters evaluated are described in Table 4. The meta-analysis demonstrated significant improvement in Qmax and urine volume, but was not able to demonstrate benefit in relation to maximum cystometric capacity and post-void residue, as shown in figures (supplementary file) and described below:

**•Maximum flow rate (Qmax)**: Significant increase (mean = 0.43; 95% CI: 0.10 to 0.77; I^2^ = 77%; p = 0.01).
**•Voided volume**: Significant increase (mean = 0.25; 95% CI: 0.10 to 0.40; I^2^ = 0%; p = 0.0009).
**•Cystometric capacity and post-void residual**: No significant changes (Supplementary Figs. 12–13).

**Table 4:** Pre- and post-treatment results of urodynamic parameters

Estudo	Jhang et al. 2017		Jhang et al. 2019		Jiang et al. 2022		Lee et al. 2022		Jhang et al. 2022		Jhang et al. 2023	
Parameter	Pre	Post	Pre	Post	Pre	Post	Pre	Post	Pre	Post	Pre	Post
CBC (ml)	300 ± 158	250 ± 53.5	274 ± 132	259 ± 106	277 ± 112	284 ± 115	253 ± 103	268 ± 101	271 ± 97.2	251 ± 144	-	-
Qmáx (ml/s)	10.5 ± 6.8	14.8 ± 6.7	10.6 ± 7.84	23.9 ± 12.4	10.2 ± 5.6	11.3 ± 5.2	9.83 ± 5.45	10.21 ± 5.28	10.3 ± 7.1	11.8 ± 5.9	13.17 ± 7.01	15.93 ± 10.8
PVR (ml)	81.7 ± 115	18.3 ± 17.4	66.4 ± 139	15.3 ± 17.2	53.3 ± 123.2	45.9 ± 91.1	43 ± 92.39	43.5 ± 92.3	86.4 ± 150	26.4 ± 31	22.97 ± 45.0	40.9 ± 99.5

### Adverse Events

Only one study (Jhang et al., 2017) reported complications: urinary tract infection in one patient (7%) and transient dysuria in three (20%). No major adverse events were observed.

## Discussion

This systematic review and meta-analysis evaluated the efficacy of intravesical platelet-rich plasma (PRP) injections for patients with interstitial cystitis/bladder pain syndrome (IC/BPS) refractory to conventional treatments. Six studies totaling 182 patients were included, all employing similar PRP preparation and administration protocols. The findings suggest that repeated intravesical injections of autologous PRP may represent a safe and potentially beneficial approach for reducing bladder pain, urinary frequency, and nocturia in this patient population. However, the magnitude of symptom improvement appears modest, and the absence of control groups precludes definitive conclusions regarding efficacy.

Treatment success, defined as a Global Response Assessment (GRA) score ≥ 2, ranged from 26.3% to 67.5% across studies. The meta-analysis estimated a pooled success rate of 48.3% (95% CI: 34.7–62.2), indicating meaningful symptom relief in approximately half of the patients. These results are particularly encouraging given that all participants had previously failed multiple treatment modalities.

Pain and voiding symptom scores (VAS, ICSI, ICPI, OSS) improved significantly post-treatment. Reductions in pain scores and increases in functional bladder capacity (FBC) indicate a possible biological effect of PRP through tissue regeneration and inflammation modulation, although these changes should be interpreted cautiously given the uncontrolled nature of the available studies.

However, improvements in urodynamic parameters were inconsistent. No significant changes were observed in maximum cystometric capacity (CBC) or post-void residual (PVR), suggesting that PRP may not directly influence detrusor function or neural control of voiding. Conversely, improvements in maximum flow rate (Qmax) and voided volume may reflect indirect benefits related to symptom relief.

These discrepancies between subjective and objective measures may reflect detection bias due to the lack of blinding in the included studies. Both participants and investigators were aware of the intervention, which may have influenced symptom reporting.

Our findings align with prior studies supporting the anti-inflammatory and regenerative potential of PRP in various degenerative and inflammatory conditions [16]. PRP is rich in growth factors and anti-inflammatory cytokines, promoting angiogenesis, epithelial healing, and neuropathic pain reduction [17]. Its widespread use in orthopedics and dermatology underlines its biological plausibility in treating IC/BPS.

Nevertheless, several limitations must be acknowledged. All included studies were quasi-experimental, lacking randomization and control groups, which limits the strength of conclusions. Additionally, variations in PRP preparation protocols—centrifugation parameters, platelet concentration, presence of leukocytes, and injection volumes—introduce methodological heterogeneity, as no standardized protocol for PRP preparation currently exists in urological applications [17]. Moderate to high heterogeneity was observed across several pooled outcomes (I^2^ ranging from 58 to 77%), reflecting these methodological differences as well as variations in injection technique and follow-up duration. This heterogeneity likely influenced the pooled estimates and may partially explain the variation in treatment response across studies. Furthermore, the limited number of available studies and their small sample sizes restrict the accuracy of heterogeneity assessment. Combined with the predominance of publications from a single research group, these factors increase the likelihood of publication bias and limit the external validity of the current evidence.

The hydrodistention performed alongside PRP in some studies may act as a confounder, given that it is itself a recognized treatment for IC/BPS. This concurrent intervention can temporarily improve bladder capacity and pain scores, potentially amplifying the perceived benefit of PRP**.** However, most patients had failed previous hydrodistention, suggesting a limited standalone effect in this context [18]. Still, its concomitant use should be considered when interpreting the magnitude of symptom improvement observed in these studies.

Another limitation is the predominance of studies conducted by a single research group, raising the risk of publication bias and limiting generalizability. This concentration of evidence from one center increases the likelihood that positive or favorable outcomes are overrepresented, while negative or neutral results may remain unpublished. Consequently, external validity is reduced, and the reproducibility of findings across different institutions remains uncertain**.** Moreover, short follow-up periods (typically three months) preclude conclusions on long-term efficacy and durability of response.

Patient selection criteria also varied. While all studies focused on refractory IC/BPS, some excluded patients with Hunner lesions—a subgroup known to respond differently to treatment—potentially impacting comparability.

Despite these limitations, the findings suggest that PRP is a promising therapeutic alternative for women with refractory IC/BPS, offering symptom relief where other options have failed.

Future research should focus on well-designed randomized controlled trials with larger sample sizes and longer follow-up. Standardization of PRP preparation and application protocols, as well as mechanistic studies, will be essential to optimize outcomes and identify patient subgroups who may benefit most from this therapy.

The results of this review must be interpreted with caution, as it is limited by the quasi-experimental design of the available studies, the lack of randomized controlled trials, and the variability in PRP preparation and injection protocols. The predominance of reports from a single research group and the relatively short follow-up periods also increase the risk of publication bias and reduce the generalizability of the findings.

## Conclusion

The treatment of IC/BPS with intravesical injection of PRP emerges as a potentially useful alternative for the management of refractory cases, demonstrating a tendency to reduce the main symptoms, such as pain, nocturia and urinary frequency, in addition to improving the functional capacity of the bladder. However, as all available studies are uncontrolled and non-randomized, the overall level of evidence remains low and the true efficacy of PRP cannot yet be determined. Further well-designed randomized controlled trials are required to better define its role in clinical practice.

## Supplementary Information

Below is the link to the electronic supplementary material.

Supplementary file1 (DOCX 87 KB)

Supplementary file2 (DOCX 4538 KB)

## Declarations

### Prior Presentation

This study has not been previously presented at any meeting or congress.
