# Supplementary material for: Intravesical Platelet-Rich Plasma Injection for Refractory Interstitial Cystitis/Painful Bladder Syndrome: A Systematic Review and Meta-analysis
Source: Int Urogynecol J. 2026 Apr 6;37(6):1515–22. doi: 10.1007/s00192-026-06515-9 (PMC13309483; doi:10.1007/s00192-026-06515-9)
Supplement: Supplementary file 1 — Supplementary file1 (DOCX 87 KB) [file 192_2026_6515_MOESM1_ESM.docx]

**Supplementary file 2**

**Search Strategy**

**"Cystitis, Interstitial" AND "Platelet-Rich Plasma" AND "Administration, Intravesical"**

| **Fonte** | **Estratégia** | **Nº de Artigos** | **Data** |
| --- | --- | --- | --- |
| **PUBMED** | **(((Cystitis, Interstitial[MeSH Terms]) OR ("Cystitis, Interstitial"[Title/Abstract] OR "Cystitides, Interstitial"[Title/Abstract] OR "Interstitial Cystitides"[Title/Abstract] OR "Interstitial Cystitis"[Title/Abstract] OR "Painful Bladder Syndrome"[Title/Abstract] OR "Bladder Pain Syndrome"[Title/Abstract] OR "Bladder Pain Syndromes"[Title/Abstract] OR "Pain Syndrome, Bladder"[Title/Abstract] OR "Cystitis, Chronic Interstitial"[Title/Abstract] OR "Chronic Interstitial Cystitides"[Title/Abstract] OR "Cystitides, Chronic Interstitial"[Title/Abstract] OR "Interstitial Cystitides, Chronic"[Title/Abstract] OR "Chronic Interstitial Cystitis"[Title/Abstract] OR "Interstitial Cystitis, Chronic"[Title/Abstract])) AND (((Platelet-Rich Plasma[MeSH Terms]) OR ("Platelet-Rich Plasma"[Title/Abstract] OR "Plasma, Platelet-Rich"[Title/Abstract] OR "Platelet Rich Plasma"[Title/Abstract])) OR ("thrombocyte rich plasma"[Title/Abstract]))) AND (((Administration, Intravesical[MeSH Terms]) OR ("Administration, Intravesical"[Title/Abstract] OR "Intravesical Drug Administration"[Title/Abstract] OR "Drug Administration, Bladder"[Title/Abstract] OR "Administration, Intravesical Drug"[Title/Abstract] OR "Administrations, Intravesical Drug"[Title/Abstract] OR "Drug Administration, Intravesical"[Title/Abstract] OR "Drug Administrations, Intravesical"[Title/Abstract] OR "Intravesical Drug Administrations"[Title/Abstract] OR "Intravesical Administration"[Title/Abstract] OR "Administrations, Intravesical"[Title/Abstract] OR "Intravesical Administrations"[Title/Abstract] OR "Bladder Drug Administration"[Title/Abstract] OR "Administration, Bladder Drug"[Title/Abstract] OR "Administrations, Bladder Drug"[Title/Abstract] OR "Bladder Drug Administrations"[Title/Abstract] OR "Drug Administrations, Bladder"[Title/Abstract] OR "Instillation, Bladder"[Title/Abstract] OR "Bladder Instillation"[Title/Abstract] OR "Bladder Instillations"[Title/Abstract] OR "Instillations, Bladder"[Title/Abstract] OR "Intravesical Instillation"[Title/Abstract] OR "Instillation, Intravesical"[Title/Abstract] OR "Instillations, Intravesical"[Title/Abstract] OR "Intravesical Instillations"[Title/Abstract] OR "Intravesical Injection"[Title/Abstract] OR "Injections, Intravesical"[Title/Abstract] OR "Injection, Intravesical"[Title/Abstract] OR "Intravesical Injections"[Title/Abstract])) OR ("bladder drug administration"[Title/Abstract] OR "bladder drug delivery"[Title/Abstract] OR "intra-vesical administration"[Title/Abstract] OR "intra-vesical drug administration"[Title/Abstract] OR "intra-vesical instillation"[Title/Abstract] OR "intravesical application"[Title/Abstract] OR "intravesical delivery"[Title/Abstract] OR "intravesical dose"[Title/Abstract] OR "intravesical drug delivery"[Title/Abstract] OR "intravesical infusion"[Title/Abstract] OR "intravesical injection"[Title/Abstract] OR "intravesical medication"[Title/Abstract] OR "intravesical therapy"[Title/Abstract] OR "intravesical treatment"[Title/Abstract]))** | **16** | **12/07/2023** |
| **PUBMED**  **PMC** | **(((Cystitis, Interstitial[MeSH Terms]) OR ("Cystitis, Interstitial"[Title/Abstract] OR "Cystitides, Interstitial"[Title/Abstract] OR "Interstitial Cystitides"[Title/Abstract] OR "Interstitial Cystitis"[Title/Abstract] OR "Painful Bladder Syndrome"[Title/Abstract] OR "Bladder Pain Syndrome"[Title/Abstract] OR "Bladder Pain Syndromes"[Title/Abstract] OR "Pain Syndrome, Bladder"[Title/Abstract] OR "Cystitis, Chronic Interstitial"[Title/Abstract] OR "Chronic Interstitial Cystitides"[Title/Abstract] OR "Cystitides, Chronic Interstitial"[Title/Abstract] OR "Interstitial Cystitides, Chronic"[Title/Abstract] OR "Chronic Interstitial Cystitis"[Title/Abstract] OR "Interstitial Cystitis, Chronic"[Title/Abstract])) AND (((Platelet-Rich Plasma[MeSH Terms]) OR ("Platelet-Rich Plasma"[Title/Abstract] OR "Plasma, Platelet-Rich"[Title/Abstract] OR "Platelet Rich Plasma"[Title/Abstract])) OR ("thrombocyte rich plasma"[Title/Abstract]))) AND (((Administration, Intravesical[MeSH Terms]) OR ("Administration, Intravesical"[Title/Abstract] OR "Intravesical Drug Administration"[Title/Abstract] OR "Drug Administration, Bladder"[Title/Abstract] OR "Administration, Intravesical Drug"[Title/Abstract] OR "Administrations, Intravesical Drug"[Title/Abstract] OR "Drug Administration, Intravesical"[Title/Abstract] OR "Drug Administrations, Intravesical"[Title/Abstract] OR "Intravesical Drug Administrations"[Title/Abstract] OR "Intravesical Administration"[Title/Abstract] OR "Administrations, Intravesical"[Title/Abstract] OR "Intravesical Administrations"[Title/Abstract] OR "Bladder Drug Administration"[Title/Abstract] OR "Administration, Bladder Drug"[Title/Abstract] OR "Administrations, Bladder Drug"[Title/Abstract] OR "Bladder Drug Administrations"[Title/Abstract] OR "Drug Administrations, Bladder"[Title/Abstract] OR "Instillation, Bladder"[Title/Abstract] OR "Bladder Instillation"[Title/Abstract] OR "Bladder Instillations"[Title/Abstract] OR "Instillations, Bladder"[Title/Abstract] OR "Intravesical Instillation"[Title/Abstract] OR "Instillation, Intravesical"[Title/Abstract] OR "Instillations, Intravesical"[Title/Abstract] OR "Intravesical Instillations"[Title/Abstract] OR "Intravesical Injection"[Title/Abstract] OR "Injections, Intravesical"[Title/Abstract] OR "Injection, Intravesical"[Title/Abstract] OR "Intravesical Injections"[Title/Abstract])) OR ("bladder drug administration"[Title/Abstract] OR "bladder drug delivery"[Title/Abstract] OR "intra-vesical administration"[Title/Abstract] OR "intra-vesical drug administration"[Title/Abstract] OR "intra-vesical instillation"[Title/Abstract] OR "intravesical application"[Title/Abstract] OR "intravesical delivery"[Title/Abstract] OR "intravesical dose"[Title/Abstract] OR "intravesical drug delivery"[Title/Abstract] OR "intravesical infusion"[Title/Abstract] OR "intravesical injection"[Title/Abstract] OR "intravesical medication"[Title/Abstract] OR "intravesical therapy"[Title/Abstract] OR "intravesical treatment"[Title/Abstract]))** | **06** | **12/07** |
| BVS / BIREME | ("Cystitis, Interstitial" OR "Cystitides, Interstitial" OR "Interstitial Cystitides" OR "Interstitial Cystitis" OR "Painful Bladder Syndrome" OR "Bladder Pain Syndrome" OR "Bladder Pain Syndromes" OR "Pain Syndrome, Bladder" OR "Cystitis, Chronic Interstitial" OR "Chronic Interstitial Cystitides" OR "Cystitides, Chronic Interstitial" OR "Interstitial Cystitides, Chronic" OR "Chronic Interstitial Cystitis" OR "Interstitial Cystitis, Chronic") AND ("Platelet-Rich Plasma" OR "Plasma, Platelet-Rich" OR "Platelet Rich Plasma" OR "thrombocyte rich plasma") AND ("Administration, Intravesical" OR "Intravesical Drug Administration" OR "Drug Administration, Bladder" OR "Administration, Intravesical Drug" OR "Administrations, Intravesical Drug" OR "Drug Administration, Intravesical" OR "Drug Administrations, Intravesical" OR "Intravesical Drug Administrations" OR "Intravesical Administration" OR "Administrations, Intravesical" OR "Intravesical Administrations" OR "Bladder Drug Administration" OR "Administration, Bladder Drug" OR "Administrations, Bladder Drug" OR "Bladder Drug Administrations" OR "Drug Administrations, Bladder" OR "Instillation, Bladder" OR "Bladder Instillation" OR "Bladder Instillations" OR "Instillations, Bladder" OR "Intravesical Instillation" OR "Instillation, Intravesical" OR "Instillations, Intravesical" OR "Intravesical Instillations" OR "Intravesical Injection" OR "Injections, Intravesical" OR "Injection, Intravesical" OR "Intravesical Injections" OR "bladder drug administration" OR "bladder drug delivery" OR "intra-vesical administration" OR "intra-vesical drug administration" OR "intra-vesical instillation" OR "intravesical application" OR "intravesical delivery" OR "intravesical dose" OR "intravesical drug delivery" OR "intravesical infusion" OR "intravesical injection" OR "intravesical medication" OR "intravesical therapy" OR "intravesical treatment" ) | 16 |  |
| **SCOPUS** | ( TITLE-ABS-KEY ( "Cystitis, Interstitial" OR "Cystitides, Interstitial" OR "Interstitial Cystitides" OR "Interstitial Cystitis" OR "Painful Bladder Syndrome" OR "Bladder Pain Syndrome" OR "Bladder Pain Syndromes" OR "Pain Syndrome, Bladder" OR "Cystitis, Chronic Interstitial" OR "Chronic Interstitial Cystitides" OR "Cystitides, Chronic Interstitial" OR "Interstitial Cystitides, Chronic" OR "Chronic Interstitial Cystitis" OR "Interstitial Cystitis, Chronic" ) ) AND ( ( TITLE-ABS-KEY ( "Platelet-Rich Plasma" OR "Plasma, Platelet-Rich" OR "Platelet Rich Plasma" ) OR TITLE-ABS-KEY ( "thrombocyte rich plasma" ) ) ) AND ( ( TITLE-ABS-KEY ( "Administration, Intravesical" OR "Intravesical Drug Administration" OR "Drug Administration, Bladder" OR "Administration, Intravesical Drug" OR "Administrations, Intravesical Drug" OR "Drug Administration, Intravesical" OR "Drug Administrations, Intravesical" OR "Intravesical Drug Administrations" OR "Intravesical Administration" OR "Administrations, Intravesical" OR "Intravesical Administrations" OR "Bladder Drug Administration" OR "Administration, Bladder Drug" OR "Administrations, Bladder Drug" OR "Bladder Drug Administrations" OR "Drug Administrations, Bladder" OR "Instillation, Bladder" OR "Bladder Instillation" OR "Bladder Instillations" OR "Instillations, Bladder" OR "Intravesical Instillation" OR "Instillation, Intravesical" OR "Instillations, Intravesical" OR "Intravesical Instillations" OR "Intravesical Injection" OR "Injections, Intravesical" OR "Injection, Intravesical" OR "Intravesical Injections" ) ) OR ( TITLE-ABS-KEY ( "bladder drug administration" OR "bladder drug delivery" OR "intra-vesical administration" OR "intra-vesical drug administration" OR "intra-vesical instillation" OR "intravesical application" OR "intravesical delivery" OR "intravesical dose" OR "intravesical drug delivery" OR "intravesical infusion" OR "intravesical injection" OR "intravesical medication" OR "intravesical therapy" OR "intravesical treatment" ) ) ) | **19** |  |
| **WEB OF SCIENCE** | "Cystitis, Interstitial" OR "Cystitides, Interstitial" OR "Interstitial Cystitides" OR "Interstitial Cystitis" OR "Painful Bladder Syndrome" OR "Bladder Pain Syndrome" OR "Bladder Pain Syndromes" OR "Pain Syndrome, Bladder" OR "Cystitis, Chronic Interstitial" OR "Chronic Interstitial Cystitides" OR "Cystitides, Chronic Interstitial" OR "Interstitial Cystitides, Chronic" OR "Chronic Interstitial Cystitis" OR "Interstitial Cystitis, Chronic" (Topic) and Preprint Citation Index (Exclude – Database) AND "Platelet-Rich Plasma" OR "Plasma, Platelet-Rich" OR "Platelet Rich Plasma" (Topic) or "thrombocyte rich plasma" (Topic) and Preprint Citation Index (Exclude – Database) AND "Administration, Intravesical" OR "Intravesical Drug Administration" OR "Drug Administration, Bladder" OR "Administration, Intravesical Drug" OR "Administrations, Intravesical Drug" OR "Drug Administration, Intravesical" OR "Drug Administrations, Intravesical" OR "Intravesical Drug Administrations" OR "Intravesical Administration" OR "Administrations, Intravesical" OR "Intravesical Administrations" OR "Bladder Drug Administration" OR "Administration, Bladder Drug" OR "Administrations, Bladder Drug" OR "Bladder Drug Administrations" OR "Drug Administrations, Bladder" OR "Instillation, Bladder" OR "Bladder Instillation" OR "Bladder Instillations" OR "Instillations, Bladder" OR "Intravesical Instillation" OR "Instillation, Intravesical" OR "Instillations, Intravesical" OR "Intravesical Instillations" OR "Intravesical Injection" OR "Injections, Intravesical" OR "Injection, Intravesical" OR "Intravesical Injections" (Topic) or "bladder drug administration" OR "bladder drug delivery" OR "intra-vesical administration" OR "intra-vesical drug administration" OR "intra-vesical instillation" OR "intravesical application" OR "intravesical delivery" OR "intravesical dose" OR "intravesical drug delivery" OR "intravesical infusion" OR "intravesical injection" OR "intravesical medication" OR "intravesical therapy" OR "intravesical treatment" (Topic) and Preprint Citation Index (Exclude – Database)  LINK PERMANENTE: <https://www.webofscience.com/wos/alldb/summary/670aab4e-15af-40e5-82ac-66e3dde31c56-978a35ae/relevance/1> | **16** |  |
| **EMBASE** | 'interstitial cystitis'/syn AND 'thrombocyte rich plasma'/syn AND 'intravesical drug administration'/syn | 19 |  |
| **COCHRANE LIBRARY** | MeSH descriptor: [Cystitis, Interstitial] explode all trees OR ("Cystitis, Interstitial" OR "Cystitides, Interstitial" OR "Interstitial Cystitides" OR "Interstitial Cystitis" OR "Painful Bladder Syndrome" OR "Bladder Pain Syndrome" OR "Bladder Pain Syndromes" OR "Pain Syndrome, Bladder" OR "Cystitis, Chronic Interstitial" OR "Chronic Interstitial Cystitides" OR "Cystitides, Chronic Interstitial" OR "Interstitial Cystitides, Chronic" OR "Chronic Interstitial Cystitis" OR "Interstitial Cystitis, Chronic"):ti,ab,kw AND MeSH descriptor: [Platelet-Rich Plasma] explode all trees OR ("Platelet-Rich Plasma" OR "Plasma, Platelet-Rich" OR "Platelet Rich Plasma"):ti,ab,kw OR ("thrombocyte rich plasma"):ti,ab,kw AND MeSH descriptor: [Administration, Intravesical] explode all trees OR ("Administration, Intravesical" OR "Intravesical Drug Administration" OR "Drug Administration, Bladder" OR "Administration, Intravesical Drug" OR "Administrations, Intravesical Drug" OR "Drug Administration, Intravesical" OR "Drug Administrations, Intravesical" OR "Intravesical Drug Administrations" OR "Intravesical Administration" OR "Administrations, Intravesical" OR "Intravesical Administrations" OR "Bladder Drug Administration" OR "Administration, Bladder Drug" OR "Administrations, Bladder Drug" OR "Bladder Drug Administrations" OR "Drug Administrations, Bladder" OR "Instillation, Bladder" OR "Bladder Instillation" OR "Bladder Instillations" OR "Instillations, Bladder" OR "Intravesical Instillation" OR "Instillation, Intravesical" OR "Instillations, Intravesical" OR "Intravesical Instillations" OR "Intravesical Injection" OR "Injections, Intravesical" OR "Injection, Intravesical" OR "Intravesical Injections"):ti,ab,kw OR ("bladder drug administration" OR "bladder drug delivery" OR "intra-vesical administration" OR "intra-vesical drug administration" OR "intra-vesical instillation" OR "intravesical application" OR "intravesical delivery" OR "intravesical dose" OR "intravesical drug delivery" OR "intravesical infusion" OR "intravesical injection" OR "intravesical medication" OR "intravesical therapy" OR "intravesical treatment"):ti,ab,kw | 03 | 12/07/2023 |
| **PROQUEST** | (abstract("Cystitis, Interstitial" OR "Cystitides, Interstitial" OR "Interstitial Cystitides" OR "Interstitial Cystitis" OR "Painful Bladder Syndrome" OR "Bladder Pain Syndrome" OR "Bladder Pain Syndromes" OR "Pain Syndrome, Bladder" OR "Cystitis, Chronic Interstitial" OR "Chronic Interstitial Cystitides" OR "Cystitides, Chronic Interstitial" OR "Interstitial Cystitides, Chronic" OR "Chronic Interstitial Cystitis" OR "Interstitial Cystitis, Chronic") OR title("Cystitis, Interstitial" OR "Cystitides, Interstitial" OR "Interstitial Cystitides" OR "Interstitial Cystitis" OR "Painful Bladder Syndrome" OR "Bladder Pain Syndrome" OR "Bladder Pain Syndromes" OR "Pain Syndrome, Bladder" OR "Cystitis, Chronic Interstitial" OR "Chronic Interstitial Cystitides" OR "Cystitides, Chronic Interstitial" OR "Interstitial Cystitides, Chronic" OR "Chronic Interstitial Cystitis" OR "Interstitial Cystitis, Chronic")) AND (abstract("Platelet-Rich Plasma" OR "Plasma, Platelet-Rich" OR "Platelet Rich Plasma") OR title("Platelet-Rich Plasma" OR "Plasma, Platelet-Rich" OR "Platelet Rich Plasma") OR abstract("thrombocyte rich plasma") OR title("thrombocyte rich plasma")) AND (abstract("Administration, Intravesical" OR "Intravesical Drug Administration" OR "Drug Administration, Bladder" OR "Administration, Intravesical Drug" OR "Administrations, Intravesical Drug" OR "Drug Administration, Intravesical" OR "Drug Administrations, Intravesical" OR "Intravesical Drug Administrations" OR "Intravesical Administration" OR "Administrations, Intravesical" OR "Intravesical Administrations" OR "Bladder Drug Administration" OR "Administration, Bladder Drug" OR "Administrations, Bladder Drug" OR "Bladder Drug Administrations" OR "Drug Administrations, Bladder" OR "Instillation, Bladder" OR "Bladder Instillation" OR "Bladder Instillations" OR "Instillations, Bladder" OR "Intravesical Instillation" OR "Instillation, Intravesical" OR "Instillations, Intravesical" OR "Intravesical Instillations" OR "Intravesical Injection" OR "Injections, Intravesical" OR "Injection, Intravesical" OR "Intravesical Injections") OR title("Administration, Intravesical" OR "Intravesical Drug Administration" OR "Drug Administration, Bladder" OR "Administration, Intravesical Drug" OR "Administrations, Intravesical Drug" OR "Drug Administration, Intravesical" OR "Drug Administrations, Intravesical" OR "Intravesical Drug Administrations" OR "Intravesical Administration" OR "Administrations, Intravesical" OR "Intravesical Administrations" OR "Bladder Drug Administration" OR "Administration, Bladder Drug" OR "Administrations, Bladder Drug" OR "Bladder Drug Administrations" OR "Drug Administrations, Bladder" OR "Instillation, Bladder" OR "Bladder Instillation" OR "Bladder Instillations" OR "Instillations, Bladder" OR "Intravesical Instillation" OR "Instillation, Intravesical" OR "Instillations, Intravesical" OR "Intravesical Instillations" OR "Intravesical Injection" OR "Injections, Intravesical" OR "Injection, Intravesical" OR "Intravesical Injections") OR abstract("bladder drug administration" OR "bladder drug delivery" OR "intra-vesical administration" OR "intra-vesical drug administration" OR "intra-vesical instillation" OR "intravesical application" OR "intravesical delivery" OR "intravesical dose" OR "intravesical drug delivery" OR "intravesical infusion" OR "intravesical injection" OR "intravesical medication" OR "intravesical therapy" OR "intravesical treatment") OR title("bladder drug administration" OR "bladder drug delivery" OR "intra-vesical administration" OR "intra-vesical drug administration" OR "intra-vesical instillation" OR "intravesical application" OR "intravesical delivery" OR "intravesical dose" OR "intravesical drug delivery" OR "intravesical infusion" OR "intravesical injection" OR "intravesical medication" OR "intravesical therapy" OR "intravesical treatment")) | 08 |  |
| **TOTAL** |  | 106 |  |
| **TOTAL DUPLICATE REFERENCES** |  | 78 |  |
| **TOTAL AFTER DUPLICATE EXCLUSION** |  | 28 |  |
