# Supplementary material for: Intravesical Platelet-Rich Plasma Injection for Refractory Interstitial Cystitis/Painful Bladder Syndrome: A Systematic Review and Meta-analysis
Source: Int Urogynecol J. 2026 Apr 6;37(6):1515–22. doi: 10.1007/s00192-026-06515-9 (PMC13309483; doi:10.1007/s00192-026-06515-9)
Supplement: Supplementary file 2 — Supplementary file2 (DOCX 4538 KB) [file 192_2026_6515_MOESM2_ESM.docx]

**Supplementary File 1**

1. **Supplementary Figures**

**Fig 1.** *Visual Analog Scale*Click or tap here to enter text.

**Fig 2**. *O’Leary-Sant Score (OSS) ; Interstitial Cystitis Symptom Index (ICSI) ; Interstitial Cystitis Problem Index (ICPI)*

**Fig 3.** Traffic light chart: risk of bias

**Fig 4.** Risk of bias graph

**Fig 5**. Forest plot that demonstrates a reduction in the ICSI score after treatment.

**Fig 6.** Forest plot that demonstrates a reduction in the ICPI score after treatment.

**Fig 7.** Forest plot that demonstrates a reduction in the OSS score after treatment.

**Fig 8.** Forest plot that demonstrates a reduction in VAS after treatment.

**Fig 9.** Forest plot that demonstrates reduced urinary frequency.

**Fig 10.** Forest plot that demonstrates a reduction in nocturia

**Fig 11.** Forest plot that demonstrates increased bladder functional capacity

**Fig 12**. Forest plot that demonstrates that there was no significant effect of the treatment on the maximum cystometric capacity.

**Fig 13.** Forest plot that demonstrates that there was no significant effect of the treatment on post-void residual.

**Fig 14.** Forest plot that demonstrates an increase in Qmax after treatment.

**Fig 15.** Forest plot that demonstrates an increase in urine volume after treatment.


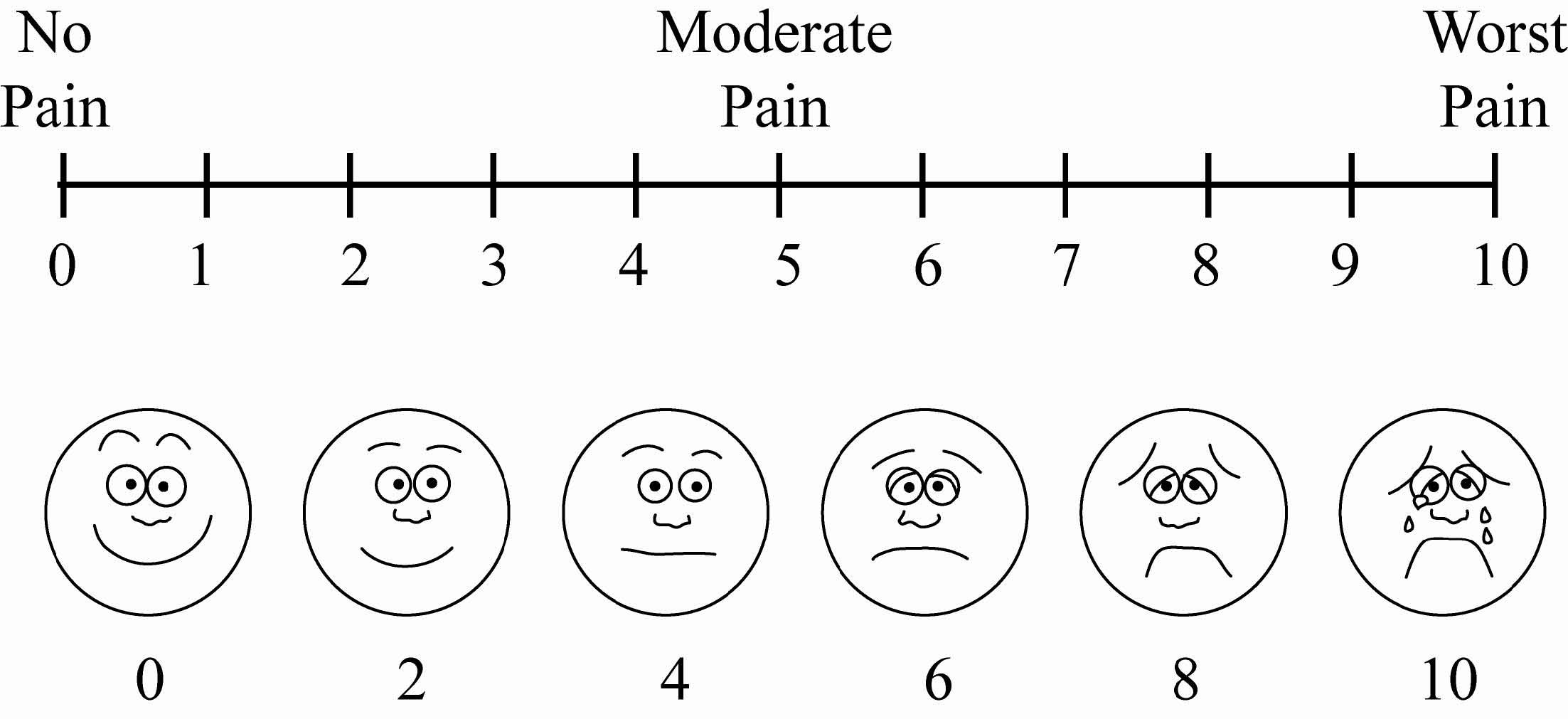


**Fig 1.** *Visual Analog Scale*Click or tap here to enter text.


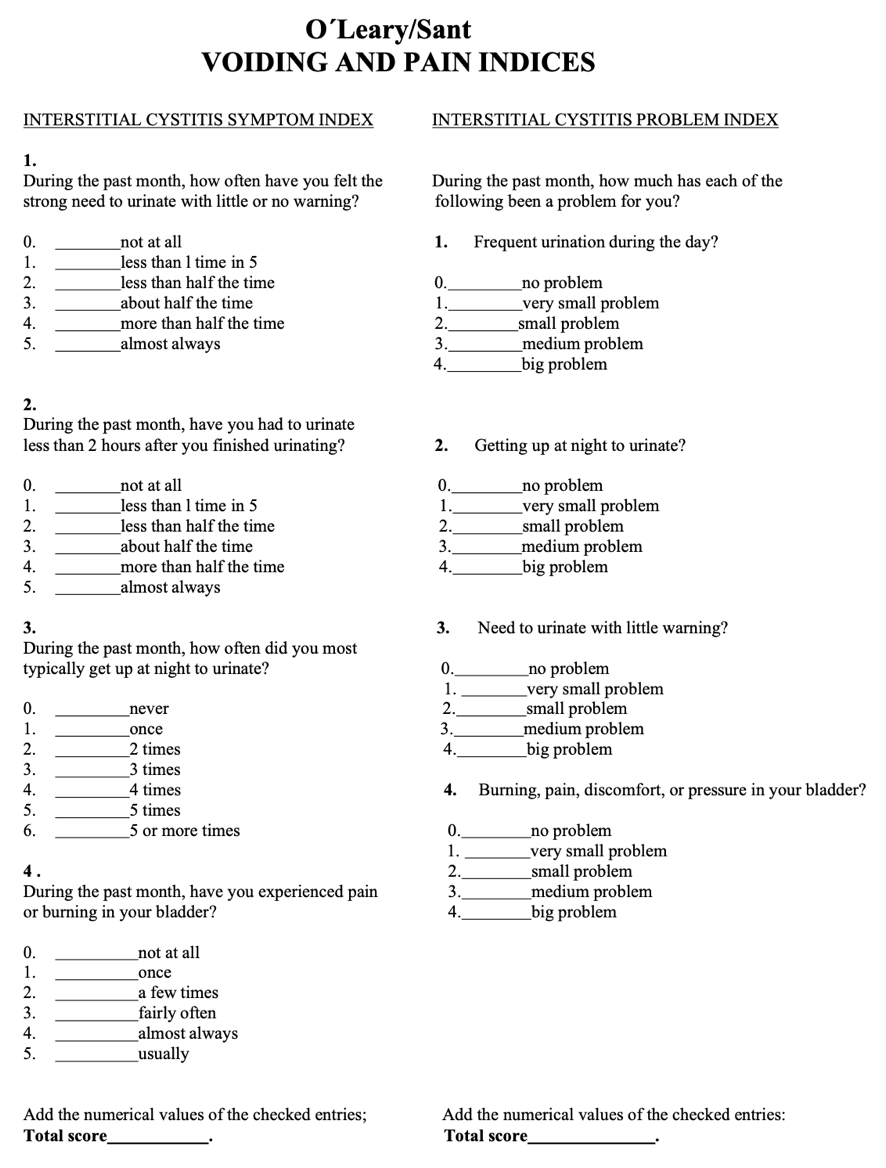


**Fig 2**. *O’Leary-Sant Score (OSS) ; Interstitial Cystitis Symptom Index (ICSI) ; Interstitial Cystitis Problem Index (ICPI)*


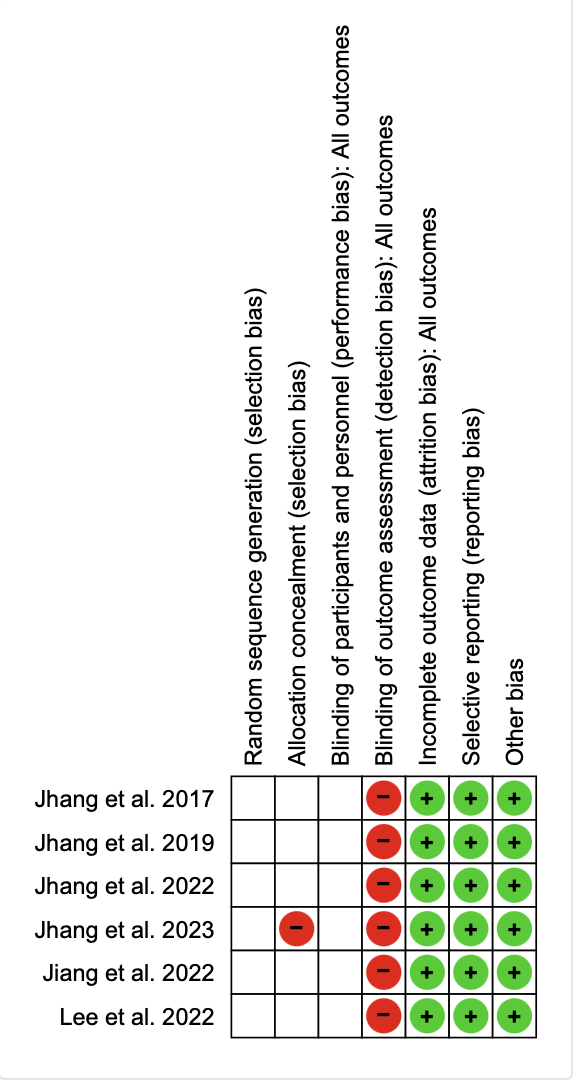


**Fig 3.** Traffic light chart: risk of bias


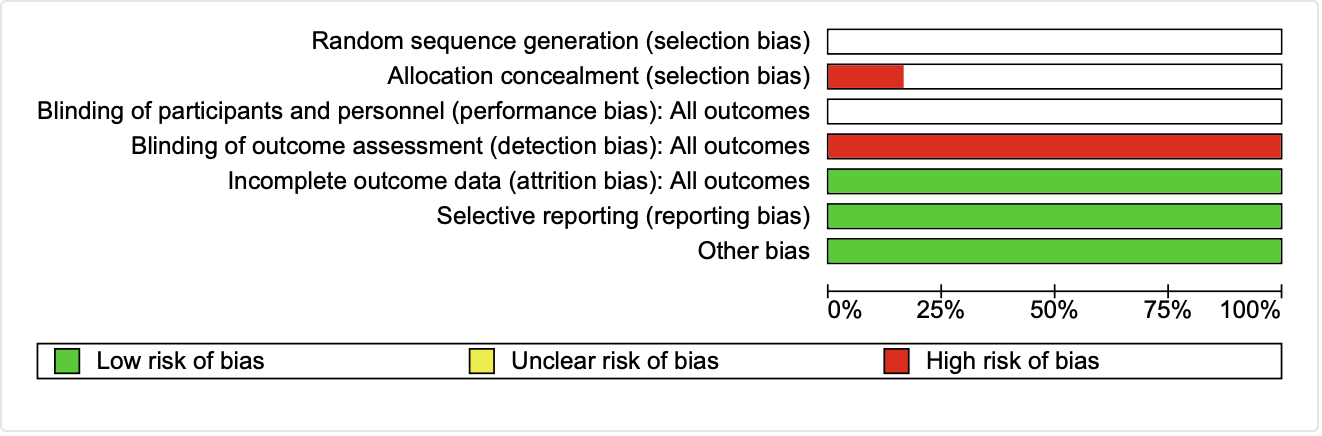


**Fig 4.** Risk of bias graph


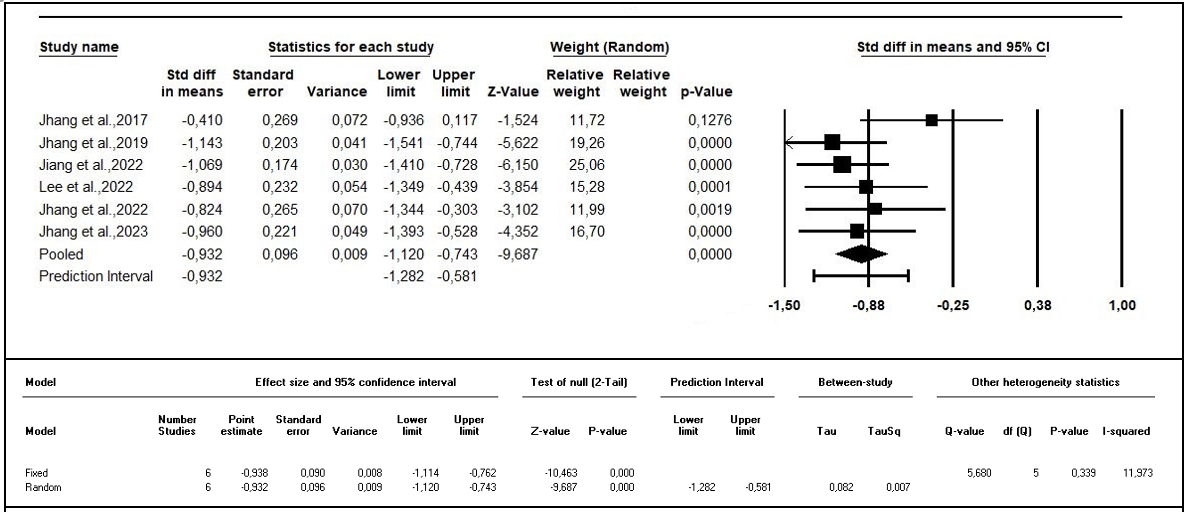


**Fig 5**. Forest plot that demonstrates a reduction in the ICSI score after treatment.


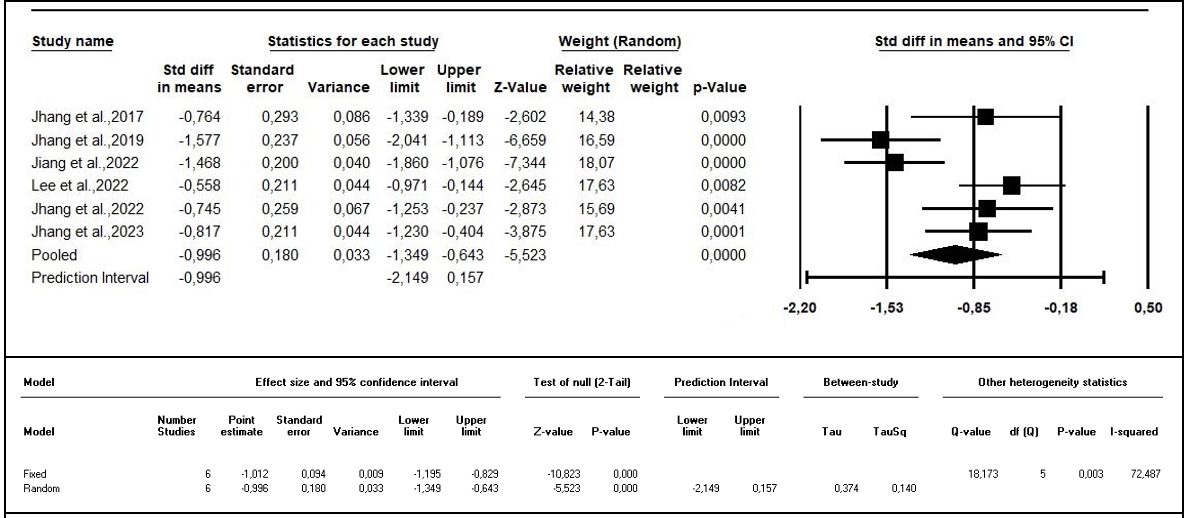


**Fig 6.** Forest plot that demonstrates a reduction in the ICPI score after treatment.


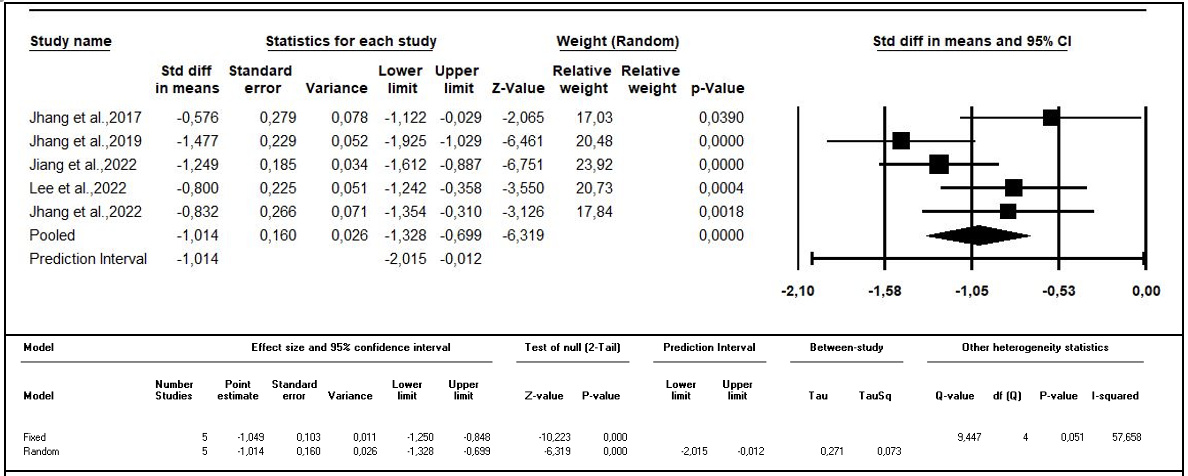


**Fig 7.** Forest plot that demonstrates a reduction in the OSS score after treatment.


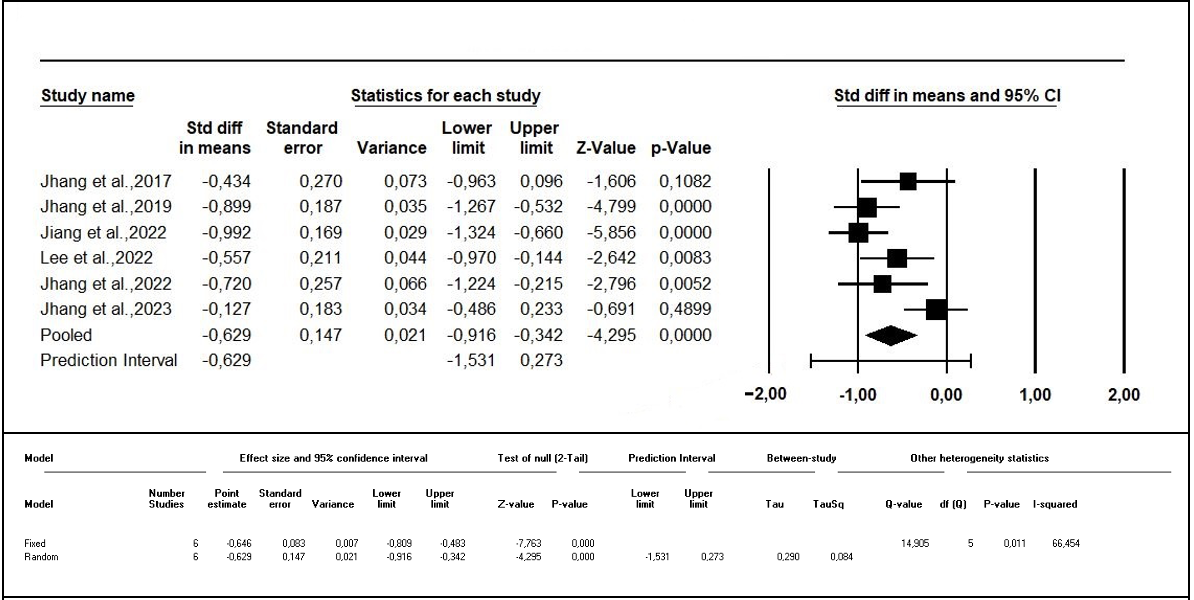


**Fig 8.** Forest plot that demonstrates a reduction in VAS after treatment.


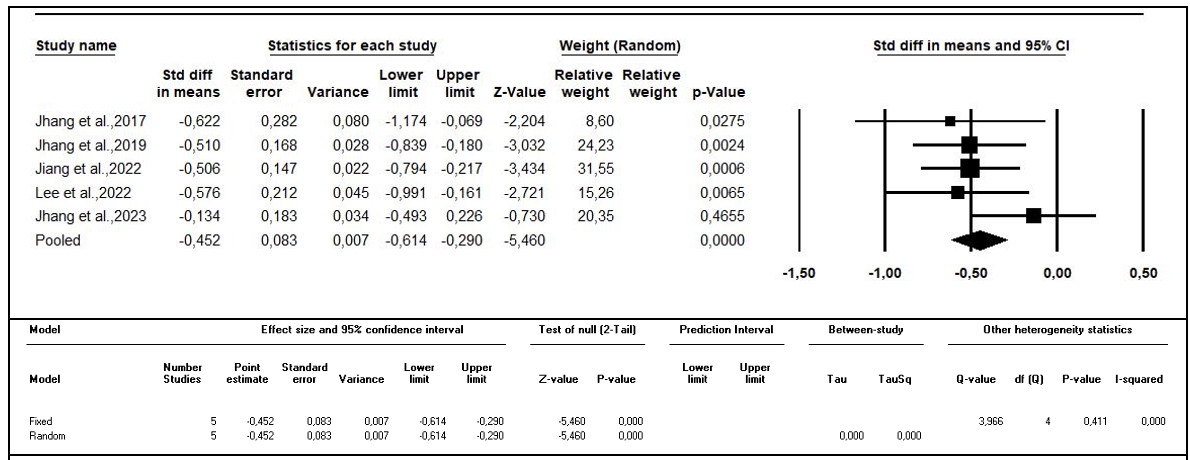


**Fig 9.** Forest plot that demonstrates reduced urinary frequency.


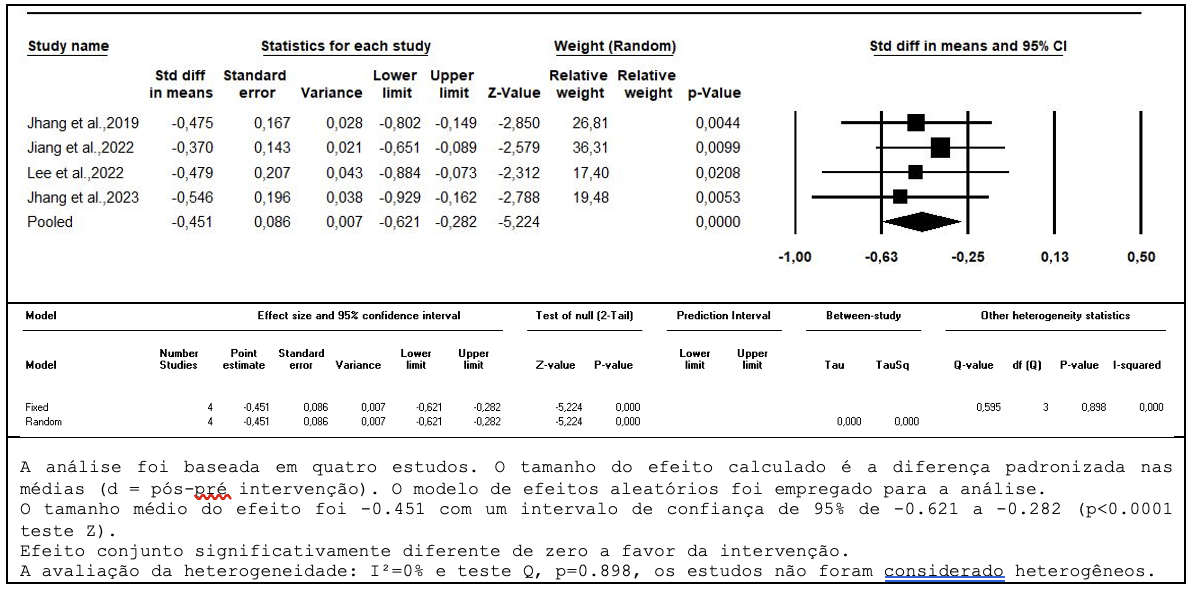


**Fig 10.** Forest plot that demonstrates a reduction in nocturia


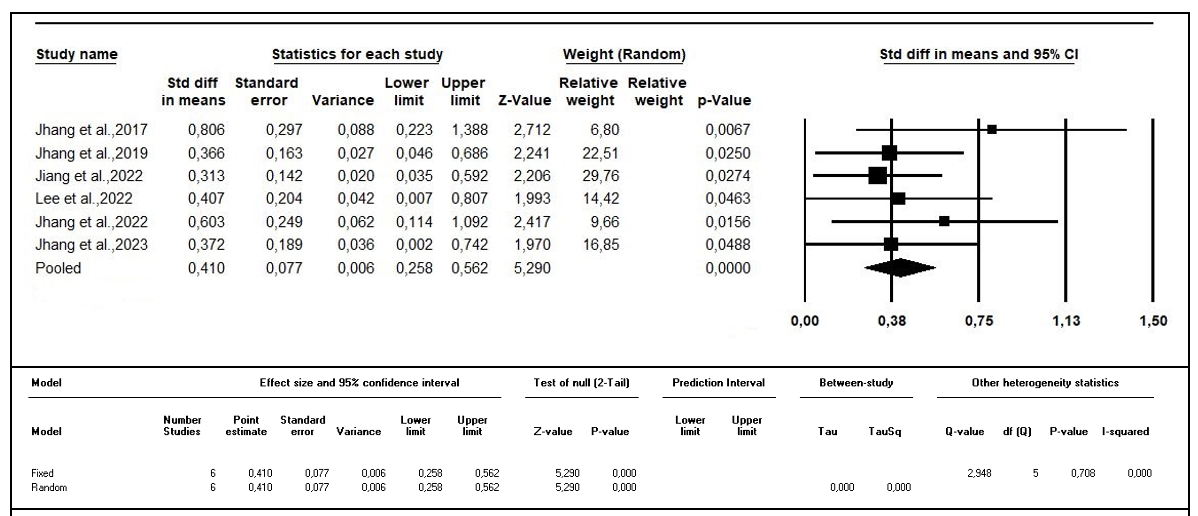


**Fig 11.** Forest plot that demonstrates increased bladder functional capacity


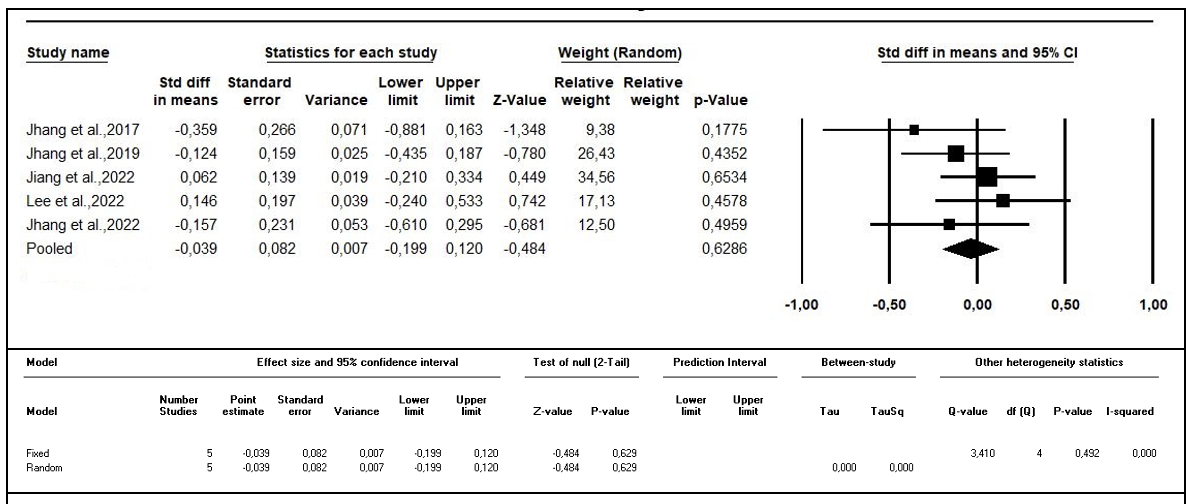


**Fig 12**. Forest plot that demonstrates that there was no significant effect of the treatment on the maximum cystometric capacity.


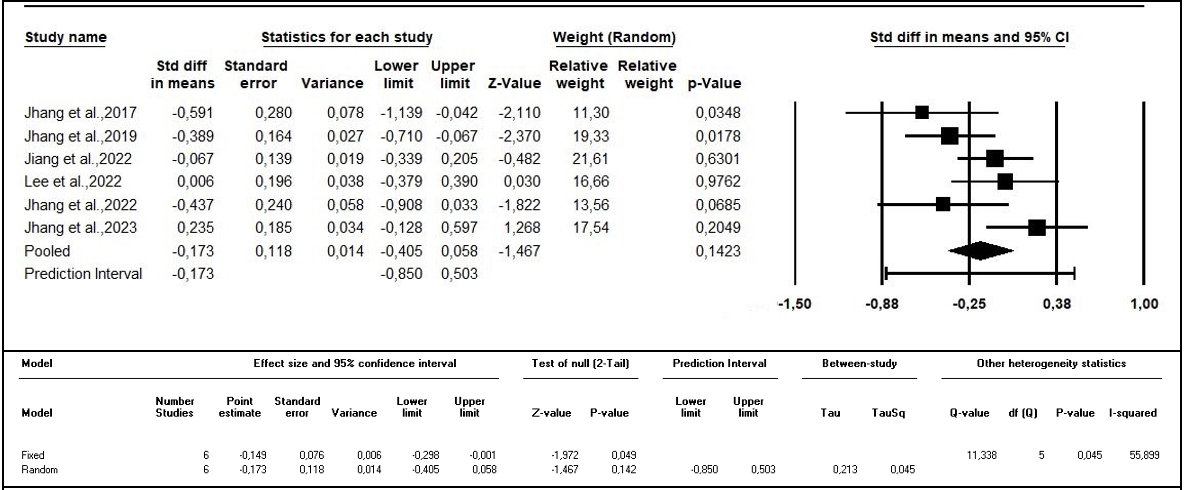
 **Fig 13.** Forest plot that demonstrates that there was no significant effect of the treatment on post-void residual.


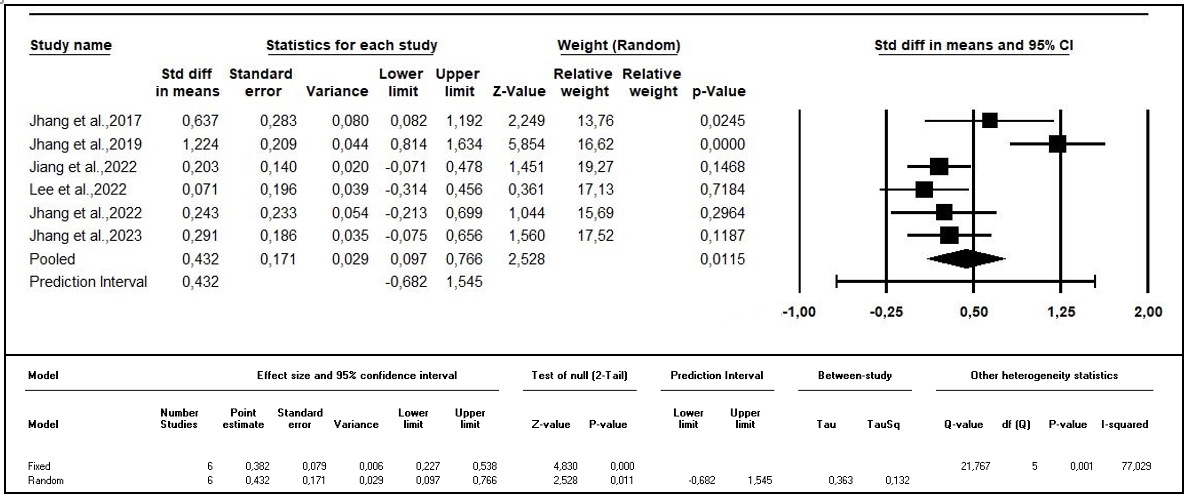


**Fig 14.** Forest plot that demonstrates an increase in Qmax after treatment.


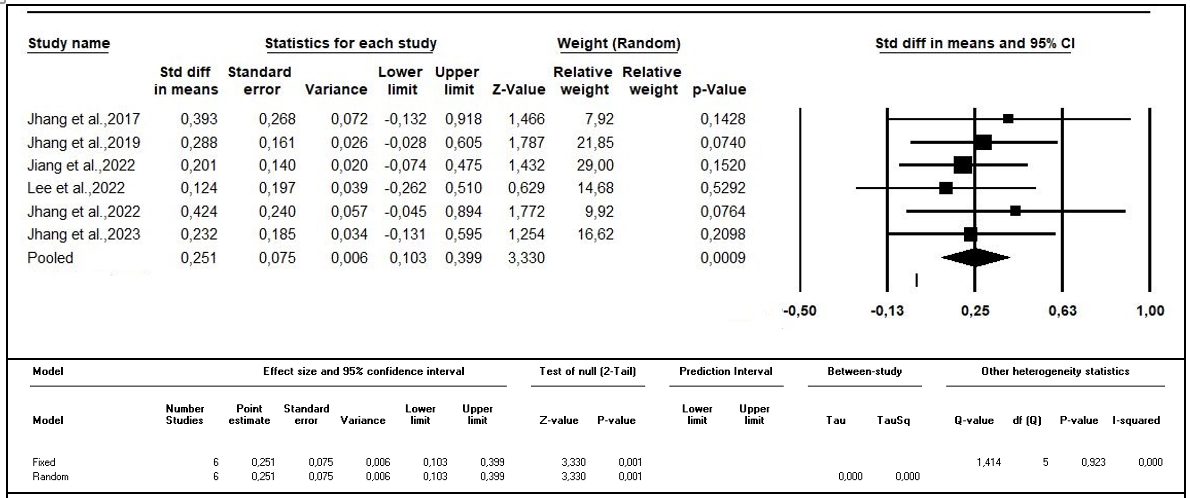


**Fig 15.** Forest plot that demonstrates an increase in urine volume after treatment.

1. **Supplementary Tables**

**Table 1.** *Global Response Assessment* (GRA)

| **Score** | **Description** |
| --- | --- |
| **-3** | Much Worse |
| **-2** | Worse |
| **-1** | Slightly Worse |
| **0** | No Change |
| **1** | Slightly Better |
| **2** | Better |
| **3** | Much Better |
